# Institutional Analysis of the Surgical Outcomes of Cochlear Implantation in Deprived Population

**DOI:** 10.7759/cureus.31853

**Published:** 2022-11-24

**Authors:** Amit K Sharma, Mahesh Kumar, Alekh Kumar, Rakesh K Singh

**Affiliations:** 1 Otolaryngology - Head and Neck Surgery, Indira Gandhi Institute of Medical Sciences, Patna, IND

**Keywords:** kuppuswamy socioeconomic scale, posterior tympanotomy, indira gandhi institute of medical sciences, surgical complications, deafness, deprived, socio-demographic, cochlear implant

## Abstract

Background: Cochlear implantation (CI) surgeries are safe and performed successfully in many patients; however, postoperative complications still occur, which may be influenced by socioeconomic and demographic variables.

Methodology: This is a retrospective observational study of 146 adults and children with hearing loss, who had CI surgery between 2017 and 2022. This study aims to assess the frequency and nature of surgical complications in patients from a low socioeconomic background who underwent CI surgery in different geographic areas. For the analysis of data, IBM SPSS Statistics for Windows, Version 21.0 (Released 2012; IBM Corp., Armonk, New York, United States) was used.

Result: A total of 146 candidates were evaluated, out of which 82 were male (56.16%) and 64 (43.84%) were female. The age of patients at the time of surgery ranged from 1-50 years. All of the patients underwent unilateral CI. Eighteen implanted patients developed minor complications with an incidence rate of 12.31%. There were no major complications observed. Further, we did not observe any post-operative meningitis as our study group was vaccinated against pneumococcal and meningococcal diseases and *Haemophilus influenzae* type B (Hib).

Conclusion: CI is an effective and safe surgical procedure for the treatment and rehabilitation of people who are deaf. It is possible to avoid complications by using meticulous surgical methods and regular post-operative monitoring to identify and treat any issues as soon as possible, regardless of socioeconomic status.

## Introduction

One of the most avoidable forms of disability seen all over the globe is hearing loss, which may refer to either a partial or complete inability to hear [1]. It is the third most common chronic condition in children and adults and has profound health-related implications [2]. The World Health Organization (WHO) projected that around 466 million individuals worldwide were impacted by hearing loss in 2018, and this number is anticipated to continue in the coming years [3]. Furthermore, more than 80% of people with a hearing disability reside in developing countries. Comparing early identification, timely intervention, and rehabilitation of people with hearing disabilities between developed and developing countries, there is a huge gap based on socioeconomic levels, educational status, healthcare infrastructure, government or private-based healthcare programs, and disease database national registry system, data analysis, outcome analysis, and research and further healthcare planning complicate the entire intervention and rehabilitation [4]. Hence, it is imperative to analyze the intervention and outcome of any intervention in diverse conditions, particularly the surgical efficacy and outcome.

Cochlear implant (CI) is considered one of modern medicine's outstanding achievements, restoring hearing function for children and adults with severe to profound deafness who find traditional hearing aids ineffective [5]. Children with bilateral CI had a higher chance of having better listening outcomes than children with digital hearing aids in an observational study carried out in the United Kingdom by Lovett and colleagues [6]. The CI is a tiny electronic device that activates the auditory nerve directly via sensors and converts audio signals into electrical stimulation [7].

Although CI surgery has been conducted successfully on many individuals, complications related to surgical procedures still arise [8]. The implanted part of CI is believed to be a device that is placed for life and is covered by the overlaying skin and soft tissues, which are all securely adherent to the underlying bone of the skull. Hence, the course of CI surgery comes with its own unique set of complications, which may be major or minor. These complications are linked to either the complexity of the procedure itself, the implantation of a deeply positioned foreign body on the scalp, or the device's malfunction [9]. Minor problems may be addressed with conservative approaches, medicinal therapy, and mild surgical operations, but significant complications can be highly severe disorders that need extended hospitalization and surgical intervention [10-12]. The number of CI recipients is increasing substantially, so patients and physicians must be aware of the complications that can occur due to the surgical technique. A comprehensive understanding of indications, limits, and possible hazards is necessary for CI to be successful as an auditory rehabilitation.

Knowledge of the risks associated with CI is essential. Retrospective reviews are often used to investigate possible CI complications, and it is an easy and inexpensive method for evaluating various complications simultaneously. This study's primary objective was to evaluate the complications encountered in children and adults of varying age groups from an underprivileged population who underwent CI in our center to contribute more data to the existing literature. In addition, this study aimed to analyze the rate of complications to provide a base for subsequent efforts to reduce the occurrence of these issues.

## Materials and methods

This was an institutional retrospective observational study of 146 CI candidates (children and adults) operated between September 2017 and April 2022. This study aimed to assess the frequency and nature of surgical complications in low socioeconomic-background patients from different geographic areas who underwent CI surgery. The data was collected from the otorhinolaryngology department of Indira Gandhi Institute of Medical Sciences, Patna, India. Patients who met the inclusion criteria (Table 1) were included in this study. Ethical approval of the study was taken from the Institutional Ethics Committee, Indira Gandhi Institute of Medical Sciences, Patna, India (approval number: 488/IEC/IGIMS/2022 dated 01/04/2022).

**Table 1:** Inclusion and exclusion criteria

Inclusion Criteria	Exclusion Criteria
Age between 1 and 50 years	Complete cochlear agenesis and cochlear nerve aplasia
Bilateral severe to profound sensorineural hearing loss	Unilateral hearing loss
No benefit with hearing aid use for at least three months	Age less than one year or more than 50 years
Motivation and good family support	Poor motivation and family support

Patient selection for CI

A candidate's candidacy for CI was determined with the standard protocol. Patients unable to hear and speak as per their chronological age were subjected to hearing evaluation. Those patients with severe to profound hearing loss were then radiologically assessed by high-resolution computed tomography (HRCT) of the temporal bone. A multidisciplinary team did further assessment to detect other co-anomalies and determine the candidacy for CI. All patients subject to CIs were vaccinated with pneumococcal, meningococcal, and Haemophilus influenzae type B (Hib) vaccines. After meeting the candidacy criteria, the preoperative fitness clearance was taken from the anesthesia department. All those patients who met all surgery requirements were posted for CI. All procedures were carried out by a single experienced surgeon who routinely performs cochlear implants via the posterior tympanotomy round window approach. Following posterior tympanotomy, the round window niche and membrane were identified, and implantation was done using Nucleus® CI422 slim straight electrodes (Cochlear Limited, Sydney, Australia).

After proper wound closure, the intra-operative impedance measure and neural response telemetry (NRT) were performed in Auto-NRT software, software-based recordings (Custom Sound® EP 6.0, Cochlear Limited) were used to check the integrity of the electrode array, and proper placement and complete insertion of the electrode in the scala tympani of the cochlea. The postoperative management was done in the in-patient ward and was discharged based on the comfortability of the patient and the satisfaction of the surgeon. A minimum follow-up of six months was observed to analyze the complication rate.

Data collection tool

Data associated with patient demographics (age, gender, financial status, and geographical area), medical histories (duration of deafness, ear operated, and radiological findings), major and minor postoperative complications, and the duration of hospital stay were acquired by reviewing the medical records. The patients were categorized into the low, middle, and high socioeconomic groups based on the modified Kuppuswamy socioeconomic scale, considering the educational and occupational status of the head of the family and the monthly income of the family [13].

Statistical analysis

The collected data were entered into an Excel file (Microsoft Corporation, Redmond, Washington, United States) and analyzed using IBM SPSS Statistics for Windows, Version 21.0 (Released 2012; IBM Corp., Armonk, New York, United States). The frequency method was used to find the number of times the data item has occurred. The collected data were described in percentages for categorical data and mean ± SD for numerical data.

## Results

The data of 146 CI patients (children and adults) operated on between September 2017 and April 2022 were analyzed. The majority of the patients (89.73%) were in the age group of 1-10 years, 4.79% of the patients were in the age group of 11-20 years, 3.42% were in the age group of 21-30 years, and 2.05% were in the age group of 41-50 years; there were no patients in the age group of 31-40 years. Of the total sample, 56.16% of the patients were male and 43.84% were female. Most of the patients (97.26%) belonged to the lower socio-economic group, 2.05% to the middle socio-economic group, and only 0.68% to the upper socio-economic group. Of the patients, 83.56% were from rural areas and 16.44% were from urban areas. The patients included in the study were all immunized with pneumococcal, meningococcal, and Hib vaccines (Table 2).

**Table 2:** Frequency of age, sex, and socio-demographic variables

Variables	Values	Frequency	Percent
Age	1-10	131	89.73
	11-20	7	4.79
	21-30	5	3.42
	41-50	3	2.05
Sex	Female	64	43.84
	Male	82	56.16
Vaccination	Pneumococcal, meningococcal, and Hib given	146	100.0
Socio-economic condition	Lower socio-economic status	142	97.26
	Middle socio-economic status	3	2.05
	Upper socio-economic status	1	0.68
Geographic area	Rural	122	83.56
	Urban	24	16.44

The majority of the patients (89.73%) had hearing disabilities for under 1-10 years, 4.79% for 11-20 years, 3.42% for 21-30 years, and 2.05% for 41-50 years. The majority of the patients (93.15%) had been operated in the right ear, and only 5.48% of the patients had been operated in the left ear. The data of 1.37% of the patients were missing. In radiological findings, 97.26% of the patients were reported as having normal radiological findings. Other findings, such as bilateral cochlear hypoplasia, incomplete partition type II, incomplete partition type I, and hypoplastic auditory nerve, were seen in less than 1% only (Table 3).

**Table 3:** Frequency of patient conditions

Variables	Values	Frequency	Percent
Duration of Deafness	1-10	131	89.73
	11-20	7	4.79
	21-30	5	3.42
	41-50	3	2.05
Ear Operated	Missing value	2	1.37
	Left	8	5.48
	Right	136	93.15
Radiological findings	Hypoplastic auditory nerve	1	0.70
	Bilateral cochlear hypoplasia	1	0.68
	Bilateral Inner ear aplasia	1	0.68
	Inner ear aplasia	1	0.68
	Normal radiological finding	142	97.26

The majority of the patients (87.67%) had no postoperative complications, and 10.27% of the patients affected with magnet-induced boil recovered with reduced strength of the magnet. Delayed facial weakness, which occurred on the third to fifth days after surgery, recovered in eight, 10, and 15 days and affected less than 1% of the patients. After the surgery, the patients stayed in the hospital for some days. As per our convenience, the hospital stay was classified into low (two to seven days), moderate (8-12 days), and high (13-18 days) (Table 4).

**Table 4:** Postoperative complications and hospital stay

Variables	Values	Frequency	Percent
Postoperative complications	Delayed facial weakness recovered in 10 days	1	.68
	Delayed facial weakness recovered in 8 days	1	.68
	Delayed facial weakness recovered within 15 days	1	.68
	Magnet induced boil, recovered with reduced strength of magnet	15	10.27
	None	128	87.67
Hospital stays (in days)	Low (2 to 7)	76	52.05
	Moderate (8 to 12)	67	45.89
	High (13 to 18)	3	2.1

**Figure 1:**
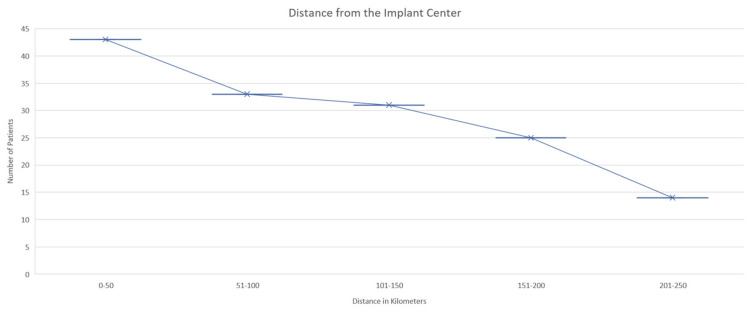
Distance from the cochlear implant center to the home of patients

The distance from the implant center to the home of the patients was categorized into five groups. Of these, 29.45% of the patients stayed within 1-50 km of the hospital, 22.60% within 51-100 km, 21.23% within 101-150 km, 9.59% within 151-200 km, and 9.59% of the patients lived within 201-250 km from the implant center (Figure 1).

## Discussion

Over the last two decades, CI has progressed through enhanced technology and techniques, resulting in fewer postoperative complications [14,15]. In addition, the companies that develop CIs make every effort to exclude the possibility of an equipment malfunction [16]. Despite all of these significant advancements, there have been reports of issues related to postoperative complications. The likelihood of the patient experiencing difficulties differs significantly depending on whether or not they are an adult or a member of the pediatric age group [17]. Our study found a 12.31% rate of minor complications (10.27% of the patients affected with magnet-induced boil and 2.04% with delayed facial weakness recovered in 8, 10, and 15 days). However, the reason for minor complications is unclear in our investigation since no significant complications were seen. According to the findings of Wilkerson et al., there was not one particular comorbidity that substantially contributed to the overall incidence of complications in the older or younger patient group [18]. According to our study's socio-demographic data, most patients (89.73 %) belonged to the age range of 1-10 years. Our findings contrast previous research, which found minor complication rates were much higher in adults, but no significant differences between adults and children. Furthermore, the radiological data of our study revealed only 2.74% concern (hypoplastic auditory nerve, bilateral cochlear hypoplasia, incomplete partition type II, and Incomplete partition type I), and no major complications were observed in our study.

It has been determined that getting an implant done before 12 months offers the most incredible benefits [19]. The average age at which a child from a rural background receives a CI is twice as high as that for a child from an urban background. This has been linked to various factors, including a lack of awareness, access to educational resources, social support, and the ability to attend appointments [20]. Regarding hearing impairment, parental education and income levels are critical factors for early intervention [21]. In our study, we found that most of the patients were aged 1 to 10 years, indicating that they received CI at a young age despite coming from rural areas (83.56 %) with lower socio-economic levels (97.26 %). On the other hand, a study in Turkey indicated that the average age at which hearing loss in children was detected was roughly 19.4 months. Moreover, the majority of the patients' residents were within 100 km (52.05 %) of the hospital, which may have made it possible to attend the hospital and encouraged the CI, as most of the rural population may not attend due to this factor, so this may be a positive factor for getting CI at the earliest stage of their life [4,20].

Facial nerve palsy after surgery is a highly uncommon condition. Edema or nerve injury can cause it to happen within the first two days, but it can also happen beyond three days, a phenomenon for which very few explanations have been suggested [22]. The passage of electric charge via the electrode, which might produce symptoms by spreading to the neighboring facial nerve, is the proposed explanation for post-cochlear implantation facial nerve stimulation. It is often found in individuals with cochlear anomalies, broken temporal bone, and otosclerosis [23]. After a CI, facial nerve palsy might occur due to the following reasons: (1) Direct facial nerve damage during surgery or indirect injury from chorda tympani nerve injury or thermal injury from drilling, (2) reactivation of herpes virus, and (3) edema and thrombus of the facial nerve owing to bacterial infection [24]. Five of 3403 (0.15 %) CI patients had facial palsy, according to Alzhrani et al. [14]. In three of the five instances, the facial nerve was damaged intraoperatively; in the other two, it was exposed but not harmed. It was found that a segment of the facial nerve was exposed during surgery in 100% of the immediate onset group and 9.5% of the delayed onset group. Further, the herpes virus' reactivation can affect the facial nerve's function. This is a well-documented risk following tympanomastoid operations, including CI. In addition, surgical operations that include significant nerve manipulation are linked to a significantly increased likelihood of viral reactivation. The delayed facial weakness that was found in our study recovered in eight days (0.68%), 10 days (0.68%), and 15 days (0.68%), which is one of the fastest rates of recovery in comparison to previous studies that discovered serious complications with extended postoperative recovery [25,26]. Around 128 (87.67%) patients did not show any postoperative complications usually associated with factors such as early age CI. Also, good surgical operations might be one of the reasons.

Facial palsy following CI surgery must be differentiated from a dangerous local infection that necessitates implant removal or reinsertion. We couldn't discover the deadly bacterial infection in our case because we started antibiotics regardless. Herpes reactivation following CI surgery might induce facial palsy, if the palsy develops within 2-14 days following surgery, herpes reactivation must be considered [27,28]. Bonkowsky et al. observed herpes simplex virus (HSV) reactivation following middle ear surgery causing delayed facial palsy [26]. Clinically verifying HSV reactivation is difficult. Seong et al. showed the usefulness of assessing antibody titers in delayed facial nerve palsy. Salvinelli et al. showed that individuals with delayed facial nerve palsy following stapes surgery had higher HSV titers. Musani et al. also recommended evaluating Bell's palsy patients' HSV-1 antibody titers [29]. After six months, 10 patients with delayed facial palsy following CI surgery were healed. According to Thom et al., the prognosis is excellent; the facial nerve palsy was resolved in three months. Patients with CI have a significantly higher risk of contracting bacterial meningitis, particularly pneumococcal meningitis [13]. Because everyone in the current study group was vaccinated against pneumococcal, meningococcal, and Hib disease, there was no instance of postoperative meningitis in our study. This is an important point that has to be underlined, and this is maybe yet another element that contributes to a lower overall rate of wound infection. These findings concord with those of Chen et al. [30].

The limitation of this study includes the sample size, which could have been more extensive, multicentral, and of multiple geographic areas. Rehabilitation was not within the scope of this article; however, it shall be incorporated in a nationwide study of deprived populations to come to a fruitful conclusion about the real efficacy and outcome of CIs in underprivileged populations.

## Conclusions

Based on the clinical experience with CI in deprived populations, our study demonstrated that the procedure is safe, with only a small number of side effects, which were manageable with non-invasive methods. It is also imperative that the complication rate is not influenced by the patient's socioeconomic and demographic factors, as shown by the findings of this study. More important is the awareness of all potential issues by the surgeon, considering these during the operation, and continuing to monitor the patient after the procedure to reduce the complication rate and to ensure safe surgery.
